# Prevalence and factors associated with irritable bowel syndrome among medical students of Karachi, Pakistan: A cross-sectional study

**DOI:** 10.1186/1756-0500-5-255

**Published:** 2012-05-24

**Authors:** Syed Saad Naeem, Efaza Umar Siddiqui, Abdul Nafey Kazi, Akhtar Amin Memon, Sumaiya Tauseeq Khan, Bilal Ahmed

**Affiliations:** 1 Medical Students, Dow Medical College, Dow University of Health Sciences, Karachi, Pakistan; 2 MSc Epidemiology and Biostatistics, Department of Medicine, Aga Khan University, Karachi, Pakistan; 3 Dow University of Health Sciences, Karachi, 74800, Pakistan

**Keywords:** Irritable bowel syndrome, Anxiety, Stress, Abdominal discomfort, Medical students

## Abstract

**Background:**

Irritable bowel syndrome (IBS) and its association with stress, has not been studied among university students in Pakistan. We investigated the prevalence and the pattern of anxiety related IBS symptoms among medical students of Karachi.

**Findings:**

An observational case–control study was carried out at three medical colleges of Karachi, Pakistan. Random sampling was done on 360 medical students. Data was collected using validated tools “Rome III Criteria” and “Generalized Anxiety Disorder Questionnaire”. Participants with IBS were diagnosed on the criteria having experienced abdominal discomfort at least 2–3 days/month associated with high level of anxiety. The apparent prevalence of IBS was found to be 28.3%, with a predominance of 87 (85.29%) females (85.29%) over males (14.71%). The psychological symptoms of anxiety were encountered in 57 (55.8%) participants with IBS, among which males were 15.7% and females 84.2% respectively.

**Conclusion:**

Students who more frequently suffer with mental stress and anxiety are more associated with IBS.

## Findings

Irritable bowel syndrome (IBS) is a functional gastrointestinal disorder illustrated by frequent abdominal pain or discomfort associated with a change in bowel habits. IBS is one of the most common diagnoses made by medical practitioners [1]. The world wide prevalence of IBS among general population ranges from 5.7% to 34% [2]. The overall prevalence of IBS in western countries as reported by various studies ranges from 17-22%. However, in Asian countries a highly variable range of prevalence has been observed i.e. 2.3-34% [3-5].

Limited data is available from Pakistan. 13% prevalence of IBS has been found in a population based study from Abbottabad, Pakistan using Rome II criteria. IBS is a multi factorial condition and is dependent on age and sex. Symptoms appear at early adulthood but decrease as age progresses. IBS prevails mostly in persons below the age of 25 years [6]. Literature reveals that IBS prevails more among females than males, although the reasons are not clear [5,7]. However, in an IBS study held in India, 7.9% males and 6.9% females [8,9].

Various diagnostic tools have been employed for detection of IBS including Manning criteria, Rome I criteria or Rome II criteria. Manning criteria appears to yield higher values compared to either the Rome I or II criteria [2]. Currently the Rome III criteria is being used more commonly [10].

Stress is a major contributing factor to IBS [11]. It has been postulated that stress stimulates colonic spasms [12]. Lately, many studies have reported that IBS is associated with elevated levels of emotional and psychological stress [13]. Some patients with IBS have reported anxiety disorders, depression as well as somatization disorder [2]. Psychosocial factors also affect the outcome of IBS in some patients. Mechanisms due to abnormal physical activity and visceral hypersensitivity are among other causative factors responsible for Irritable Bowel Syndrome [13].

It has been identified that stress is very common among medical students and is a major factor for IBS. Thus it may be possible that IBS is common among medical students. Therefore the aims of our current study were to estimate the prevalence of IBS and to estimate the association of psychological states like anxiety with IBS among medical students of Karachi, Pakistan.

It was an observational case–control study. A total of 360 medical students were included in the study which was conducted during 1^st^-31^st^, September 2010. Study population comprised of first to final year students of three large medical colleges of Karachi i.e. Aga Khan University, Dow Medical College and Sindh Medical College. Convenience non-probability sampling was done to recruit the participants aged 18 and above after administration of a written informed consent.

Data was collected using two validated questionnaires: Rome III Criteria and generalized anxiety questionnaire.

### Rome III criteria

The diagnosis of IBS was based on Rome III criteria. This is a clinical diagnostic criterion that outlines the symptoms and applies parameters such as frequency and duration allowing a more accurate diagnosis of IBS [10]. It defines IBS as recurrent abdominal pain or discomfort for at least 3 days/month during last 3 months associated with two or more of the following features:

1. Improvement with defecation. And/or

2. Onset associated with a change in frequency of stool. And/or

3. Onset associated with a change in form (appearance) of stool.

The diagnosis of IBS can reasonably be made by using the Rome III criteria as long as the patient does not have red flag symptoms like fever, vomiting, rectal bleeding, weight loss or other findings that might suggest other diagnoses.

The sensitivity of the Rome Criteria combined with the absence of red flag symptoms is 65%, specificity is 100%, the positive predictive value is 100% and the negative predictive value is 76% [14].

Severity of symptoms was obtained with a five-point scale in which 1 represented no symptoms, 2 referred to mild symptoms, 3 was for moderate symptoms, 4 represented severe symptoms and 5 reflected extremely severe symptoms that distinctly affect one’s daily life.

The subjects were further classified as having constipation predominant, diarrhea predominant, mixed or un-sub typed IBS.

### Generalized anxiety questionnaire

A thirteen-item structured generalized anxiety questionnaire was used to determine whether or not the student had a particular anxiety related disorder. The severity of anxiety was measured by calculating the scores in the questionnaire. The result fell into four grades as follows: Scores of <6 determine absence of anxiety, scores within 6–8 is low level of anxiety, 8–10 is mild level of anxiety and ≥10 determine high level of anxiety.

### Criterion for controls and cases

All those participants who were found negative for IBS using Rome III Criteria were kept as control, while those who were positive for IBS were kept as cases. Further, all the cases as well as controls were screened for anxiety levels using a generalized anxiety questionnaire.

A sample size of 360 participants was calculated using a prevalence of 34% with 80% power, 0.05% significance level, 5% bond of error and adjustment for non-response rate [15].

### Ethics statement

The study was approved by Ethical Review Committee of Aga Khan University Hospital.

### Data analysis

Statistical analysis was done by using SPSS version 16.0. Means and standard deviations were calculated for continuous variables and proportions for categorical. Chi square test was applied to derive the p-values in order to identify the significance of the results.

### Demographics

A total of 360 questionnaires were distributed, completed and analyzed with a response rate of 100%. The subjects comprised of 297 (82.5%) female and 63 (17.5%) male students with a mean age of 20.89 ± 2.8 years.

### Prevalence of IBS

Out of the 360 individuals screened for IBS using Rome III criteria, 102 cases were found to be positive, thus giving a prevalence of 28.3%. Out of the 102 positive cases, 85.29% were females (n = 87) and 14.7 % were (n = 15) males.

Age-wise distribution showed that majority of the individuals associated with IBS belonged to age-group 21–23 years (n = 62, 60.7%) followed by age-group 18–20 years (n = 34, 33.3%) (Table 1).

**Table 1:** Demographic characteristics of Medical Students

	**Irritable Bowel Syndrome by **		**Total**	**P **
	**Rome-III Criteria**		** (n = 360)**	**values**
	**Positive**	**Negative**		
	**(n = 102)**	**(n = 258)**		
**Gender:**				
Male	15 (23.8%)	48 (76.2%)	63 (100%)	0.380
Female	87 (29.3%)	210 (70.7%)	297 (100%)	
**Age group:**				
18–20 yrs	34 (22.2%)	119 (77.78%)	153 (100%)	0.014
21–23 yrs	62 (35.4%)	113 (64.6%)	175 (100%)	
24–26 yrs	06 (18.75%)	26 (81.25%)	32 (100%)	
**Medical year:**				
Non-Clinical	32 (22.2%)	112 (77.78%)	144 (100%)	0.036
Clinical	70 (32.4%)	146 (67.6%)	216 (100%)	

Of the 102 individuals found positive to be IBS, about 55% were simultaneously positive for IBS type mixed. Furthermore, 25% of the IBS-positive individuals were positive for subtype constipation (Table 2).

**Table 2:** Frequency of IBS sub-types in 102 positive cases

**Types of IBS**	**Frequency out of 102 IBS positive**		**P-values**
	**Positive**	**Negative**		
**IBS Constipation**	25 (24.5%)	77 (75.5%)	0.071	
**IBS Diarrhea**	17 (16.7%)	85 (83.3%)	0.002	
**IBS Mixed**	56 (54.9%)	45 (45.1%)	0.000	
**IBS Unknown**	04 (3.9%)	98 (96.1%)	0.000	

### Association with anxiety

Thus considering the 258 individuals found negative for IBS as our control group, about 90 of them were found to be associated with anxiety using GAQ. Thus, about 34.9% controls were associated to anxiety.

Out of our 102 cases, a total of 57 individuals were found to be positive for anxiety. Thus, about 55.8% cases were found to be associated to anxiety (Table 3).

**Table 3:** Association of Anxiety and IBS

	**Cases (IBS Positive)**	**Controls (IBS Negative)**	**P-values**
Anxiety*			
Positive	57 (55.8%)	90 (34.9%)	0.000
Negative	45 (44.2%)	168 (65.1%)	
**Total**	102 (100%)	258 (100%)	

*as assessed by Generalized Anxiety Questionnaire.

Analyzing the association between IBS subtypes and anxiety showed that about 56% of the total individuals who were IBS subtype mixed positive, were also positive for anxiety. While only 35% people who were IBS mixed type negative were anxiety positive [Table 4. Thus, IBS subtype mixed was found to have a significant Our study results show a prevalence of 28.5% IBS among medical students of Pakistan. Jafri et al. showed prevalence of IBS to be 34% among college students in 2005 [11]. Despite the difference in study groups and the fact that only two studies have been carried out on IBS prevalence among university students in Pakistan, the results of both studies were similar. One of the reasons for the slight difference in the results may be the fact that medical students are generally more aware of the IBS symptoms and can manage them more efficiently than nonmedical students. Jafri et al. also found that health seeking attitude was more common among medical than non-medical students [15].

**Table 4:** Association of Anxiety with IBS sub-types

**Types of IBS**	**Anxiety**		**Percentage**	**P-values**
	**Positive**	**Negative**		
**IBS Constipation **				
Positive	25 (37.3%)	42 (62.7%)	67 (100%)	0.516
Negative	122 (41.6%)	171 (58.4%)	293 (100%)	
**IBS Diarrhea **				
Positive	15 (45.45%)	18 (54.54%)	33 (100%)	0.571
Negative	132 (40.3%)	195 (59.6%)	327 (100%)	
**IBS Mixed **				
Positive	54 (55.7%)	43 (44.3%)	97 (100%)	0.001
Negative	93 (35.4%)	170 (64.6%)	263(100%)	
**IBS Unknown **				
Positive	53 (32.5%)	110 (67.5%)	163 (100%)	0.003
Negative	94 (47.7%)	103 (52.3%)	197 (100%)	

IBS prevalence in females was significantly higher than in males (5.8:1) in our study. It is steady with previous researches carried out especially on IBS among university students. Yan-Mei Tan et al. found that IBS among Malaysian medical students was diagnosed more commonly in females than males [16]. Lei Shen et al. showed that the prevalence of IBS among Chinese university students in men was 14.5% and 16.8% in women, indicating that female students had a larger prevalence. [2] The results of a very recent study in Japan on medical and nursing students also showed that women had a higher prevalence [17]. Prevalence in western countries also had a similar trend of women having a higher rate of IBS than men. On the contrary, a few reports from [Mumbai 8,^9^], Europe and North America point out that the rate is higher in men than women. Since our study was carried out on a small scale and there is a high ratio of females to males (297:63) studying in the setup of our study population, our results had 85.29% (87/102) females diagnosed with IBS.

The core reason behind the difference of prevalence of IBS involving the genders is uncertain. In one of the articles they were explained by differences of socio-cultural features and health care seeking behavior between men and women or true biological differences [13]. In one of the published literature, it is reported that IBS symptoms add to the distress during menstrual cycle which leads to higher number of women experiencing the symptoms more frequently [18]. Sun Young Lee et al. concluded that gastrointestinal symptoms were more frequent in women than in men regardless of the menstrual phase. Therefore, physicians should consider a gender-based approach in clinical practice [19].

Psychosocial factors have always been widely recognized and associated with IBS. Nicholl et al. had demonstrated that anxiety, depression and sleeping disorders coexist with IBS [20]. Hazlett-Stevens also found that worry, neuroticism, anxiety sensitivity and visceral anxiety are leading factors in the occurrence of IBS [21]. Reported by Lei Shen et al., 66.2% of students met the criteria for psychiatric disorder in his research conducted in 2009 [2]. Our results also established this fact with 55.8% students presenting with IBS associated with anxiety.

Medical students experience more psychological stressors due to exams and study load. Participants had a coexistence of other psychological symptoms like they easily got fatigued or had difficulty in controlling their anxiety. They had lack of concentration in different tasks. These individuals have a difficulty in controlling their anger and this interferes with their normal routine, work or university activities. The prevalence of anxiety is based on items from our structured questionnaire; a criterion is shown in the Table 5.

**Table 5:** Analysis of anxiety parameters in IBS positive (n = 102) individuals

**Anxiety Parameters:**	**Females**	**Males**	**Total**	**Percentage**
Easily fatigued in daily routine work	50	9	59	57.8%
Everyday worries keep you preoccupied	48	8	56	54.9%
Difficulty over controlling anxiety and worries	45	5	50	49%
Trouble in keeping mind on one thing	47	10	57	55.9%
Having headache frequently	45	7	52	51%
Feeling restless most of the time	30	5	35	34.3%

In our study, out of the 102 IBS positive cases, symptom subgroups analysis based on the predominant bowel habit showed that majority of these individuals had the mixed predominant subtype (54.9%) [Table 4. However, a similar study from Malaysia reported the predominant subtype to be only constipation [16]. It might be due to more stress levels in our study population or other factors contributing to a more severe form in our population.

There are a number of limitations to our present study. Firstly, our data is based on a selected group of medical students and does not signify the general population. Secondly, due to excessive workload on medical students, the specifications of questionnaires might not have been accurately filled. Thirdly, the extra stress on students having their exam may have exaggerated their gastrointestinal symptoms and psychological stressors affecting the results. Lastly the cause and effect relationship for the identified associated factors could not be established in detail.

The above facts clearly indicate high occurrence of IBS among medical students of Karachi which can be greatly detrimental. There is an immediate need to aware the students from the possible negative outcomes of this condition. Stress which is a major contributing factor in the development of IBS has to be tackled. Counseling sessions can play an important role in reducing the amount of stress on medical students.

## Abbreviation

IBS, Irritable Bowel Syndrome.

## Competing interests

The authors declare that they have no competing interests.

## Authors’ contributions

**SSN:** Wrote the article and synopsis for this research. **EUS:** Wrote the article and synopsis for this research. **STK:** Collected data and did data entry. **ANK:** Collected data and did data entry. **AAM:** Made edits and changes. **BA:** Revised and did analysis of the article. All authors read and approved the final manuscript.
